# Evaluation of residual plastic film pollution in pre-sowing cotton field using UAV imaging and semantic segmentation

**DOI:** 10.3389/fpls.2022.991191

**Published:** 2022-09-09

**Authors:** Zhiqiang Zhai, Xuegeng Chen, Ruoyu Zhang, Fasong Qiu, Qingjian Meng, Jiankang Yang, Haiyuan Wang

**Affiliations:** ^1^College of Mechanical and Electrical Engineering, Shihezi University, Shihezi, China; ^2^Key Laboratory of Northwest Agricultural Equipment, Ministry of Agriculture and Rural Affairs, Shihezi, China

**Keywords:** UAV imaging, deep learning, cotton field, residual film, pollution

## Abstract

To accurately evaluate residual plastic film pollution in pre-sowing cotton fields, a method based on modified U-Net model was proposed in this research. Images of pre-sowing cotton fields were collected using UAV imaging from different heights under different weather conditions. Residual films were manually labelled, and the degree of residual film pollution was defined based on the residual film coverage rate. The modified U-Net model for evaluating residual film pollution was built by simplifying the U-Net model framework and introducing the inception module, and the evaluation results were compared to those of the U-Net, SegNet, and FCN models. The segmentation results showed that the modified U-Net model had the best performance, with a mean intersection over union (MIOU) of 87.53%. The segmentation results on images of cloudy days were better than those on images of sunny days, with accuracy gradually decreasing with increasing image-acquiring height. The evaluation results of residual film pollution showed that the modified U-Net model outperformed the other models. The coefficient of determination(R^2^), root mean square error (RMSE), mean relative error (MRE) and average evaluation time per image of the modified U-Net model on the CPU were 0.9849, 0.0563, 5.33% and 4.85 s, respectively. The results indicate that UAV imaging combined with the modified U-Net model can accurately evaluate residual film pollution. This study provides technical support for the rapid and accurate evaluation of residual plastic film pollution in pre-sowing cotton fields.

## Introduction

Plastic film mulching is an agricultural technique that can improve soil temperature, reduce soil water loss, suppress weed growth, and improve crop water use efficiency, yield, and quality (Yan et al., 2014; Xue et al., 2017). However, much of the waste plastic film remains in the soil after harvesting. With polyethylene as raw material, plastic film is decomposed into residual film and microplastics over time under natural conditions (Qi et al., 2020; Zhang et al., 2022). However, complete decomposition of plastic film in the soil requires 200 to 400 years (He et al., 2009). The increase in residual film in the soil has brought a series of serious problems, such as soil structure damage, decreased soil quality, and crop yield loss (Dong et al., 2015).

Cotton is one of the major cash crops in the world (Akter et al., 2018; Alves et al., 2020). China is one of the world’s leading cotton growers, and Xinjiang Province has become an important region for high-quality cotton production in China. In 2021, Xinjiang’s cotton production reached 5.129 million tons, accounting for approximately 89.5 percent of China’s total cotton output. Due to the arid climate in Xinjiang, farms have used film mulching in cotton planting for a long time. However, the accumulation of plastic film waste has caused serious white pollution to farmland (Zhao et al., 2017).

Farmland residual film pollution control is a systematic project. In addition to the development of residual film recycling machines, it is of great significance to carry out efficient and accurate residual film pollution monitoring to provide reference for reducing residual film pollution in farmlands.

At present, artificial collection of residual films is mostly used for residual film pollution evaluation. For example, Zhang et al. (2016) studied the status and distribution characteristics of residual film in Xinjiang, the results indicated that the thickness of the film had significantly negative correlation with the amount of residual film. Wang et al. (2022) analyzed residual film pollution in northwest China and found that plastic debris residing in soil tend to be fragmented, which could make plastic film recovery more challenging and cause severe soil pollution. He et al. (2018) and Wang et al. (2018) used manually stratified sampling to monitor cotton fields with different duration of film mulching according to the weight and area of residual film. They found that residual film content increased year by year as the film mulching continued, and the residual film broke down and moved into the deep soil during crop cultivation. However, artificial collection of residual films, with high labour intensity and low efficiency, cannot meet the requirement for rapid monitoring of residual film pollution. Therefore, it is urgent to develop an efficient evaluation method for evaluating farmland residual film pollution at present.

With the rapid development of UAV remote sensing and deep learning technology, UAV imaging combined with semantic segmentation has been increasingly widely used in agriculture. Zhao et al. (2019) collected UAV RGB and multispectral images of rice lodging and proposed a U-shaped network-based method for rice lodging identification, finding that the Dice coefficients for RGB and multispectral images were 0.9442 and 0.9284, respectively. Zou et al. (2021) proposed a weed density evaluation method using UAV imaging and modified U-Net, and the intersection over union (IOU) was 93.40%. Li et al. (2022) proposed a method for high-density cotton yield estimation based on low-altitude UAV imaging and CD-SegNet. They found that the segmentation accuracy reached 90%, and the average error of the estimated yield was 6.2%.

In recent years, some scholars have preliminarily explored UAV imaging-based plastic film-mulched area detection and residual film identification. Zhu et al. (2019) proposed a method for extracting the plastic film-mulched area in farmlands using UAV images. Based on UAV remote sensing images, the white and black film-mulched areas in farmlands were extracted, and the accuracy reached 94.84%. Sun et al. (2018) proposed an area estimation approach for plastic film-mulched areas based on UAV images and deep learning, and five fully convolutional network (FCN) models were built by multiscale fusion, finding that the optimal identification accuracy of the FCN-4 s model was 97%. Tarantino and Figorito (2012) used the object-oriented nearest neighbour classification method to extract mulching information from aerial images. In addition, focused on farmland residual film pollution Wu et al. (2020) proposed a method for plastic film residue identification using UAV images and a segmentation algorithm. To overcome the influence of light on the accuracy of residual film identification, an impulse coupled neural network based on the S component was built, and the average identification rate was 87.49%. However, this research aimed at farmland that was not ploughed after harvesting in autumn, residual film had good continuity and low fragmentation.

It is of great significance to monitor whether the farmland reaches the qualified conditions for sowing by the rapid detection of residual film pollution in pre-sowing cotton field. Before sowing in the spring, the agricultural mulch turned into film fragments as the cotton field went through a series of operations, such as straw crushing, ploughing, and field preparation et al. Compared with plastic film mulch area detection after sowing in spring and plastic film residue detection after harvest in autumn, residual film pollution evaluation in pre-sowing cotton fields is more difficult.

Aimed at detecting residual film coverage rate in pre-sowing cotton field surface, Zhai et al. (2022) proposed a detection method based on pixel block and machine learning, however, the Mean Intersection Over Union(MIOU) was only 71.25%, and the image acquisition method was near-ground imaging, which is not convenient for rapid monitoring of residual film pollution. Therefore, this study proposed a method for residual film pollution evaluation in pre-sowing cotton fields based on UAV imaging and deep learning semantic segmentation algorithm, aiming to achieve rapid and accurate identification of residual films in pre-sowing cotton fields. This study provides a theoretical basis for further research on the rapid and accurate evaluation technology equipment for residue film pollution.

## Materials and methods

### Data acquisition

Residual film images were collected from Shihezi City, Xinjiang, China (43°26′ ~ 45°20′N, 84°58′ ~ 86°24′E, a.s.l. 450.8 M), where has a temperate continental climate. The main crops in this area were cotton, and drip irrigation - plastic film mulching has been widely adopted in cotton planting (Wang et al., 2021). The amount of mulch films (thickness: approximately 0.008 mm) used during sowing was between 75 and 120 kg·hm^−2^. After harvesting in autumn, straw return was performed after crushing, and films were recovered. Ploughing and other operations were carried out in cotton fields before sowing in spring.

**Figure 1:**
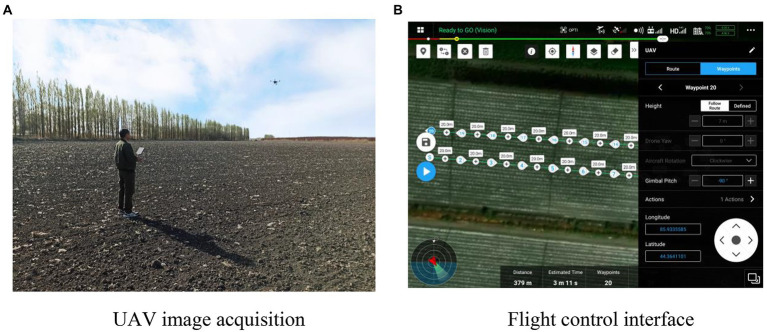
UAV image acquisition **(A)** and flight control parameters **(B)**.

In this study, UAV images of 20 residual plastic film-polluted cotton fields were collected using a DJI M200 aircraft (DJI Innovation Technology Co., Ltd., DJI-Innovations) equipped with a Zen Zenmuse X4S camera from 10:00 to 19:00 on sunny and cloudy days from April 5 to April 15, 2021. The image resolution was 5,472 × 3,078 pixels. As shown in Figure 1, the waypoint method was used for flight for image acquisition. Each cotton field had 10 flight points in a straight line, and the distance between each point was 20 m. The flight speed of the UAV was 3 m/s, the camera angle was 90°, perpendicular to the ground, and the image-acquiring height were 5, 7, and 9 M. A total of 600 images were collected. Original UAV image data distribution of residual film in cotton field is shown in Table 1. In this study, 600 images were divided into a training set (480), validation set (60), and test set (60).

**Table 1:** Original UAV image data distribution of residual film in cotton field.

	5 m	7 m	9 m	Total
Sunny	100	100	100	300
Cloudy	100	100	100	300
Total	200	200	200	600

### Image labelling and data enhancement

**Figure 2:**
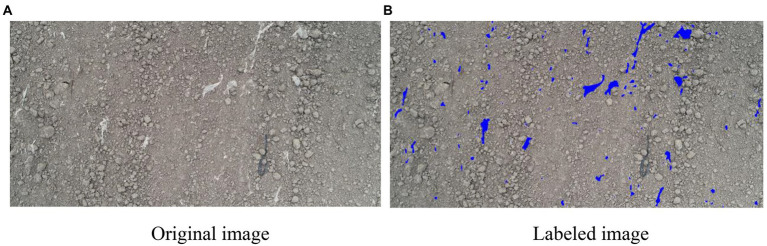
Image labelling: **(A)** Original image; **(B)** Labeled image.

The images were manually annotated using Adobe Photoshop CS5 (Adobe Systems Inc., United States), and all residual films were manually annotated and filled with blue color. Then, the threshold segmentation method was used for binarization. Residual film pixels were labelled as 1, and background pixels such as soil were labelled as 0. The annotation results are shown in Figure 2.

As the original images were too large to directly use for training, to accelerate the model calculation, the image resolution was resized to 1,200 × 600 pixels. In addition, the training set data were enhanced in the process of model training. In each epoch of training, random cutting (size: 1024 × 512 pixels), random flipping (left and right), random flipping (up and down), and brightness adjustment were used for data enhancement. Each training epoch obtained 480 new training data, and 55 epochs of training were conducted. Finally, a total of 26,400 enhanced images were obtained and used for training.

### Residual film images segmentation network structure

**Figure 3:**
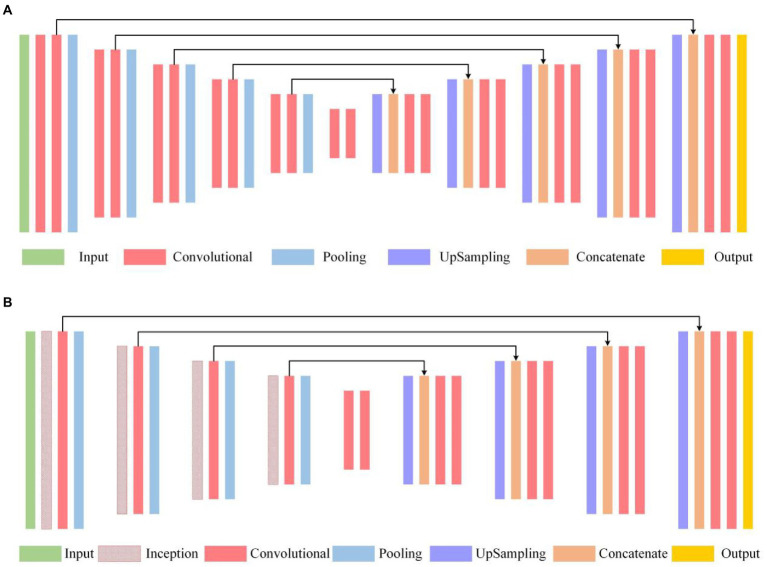
Network structure: **(A)** Structure of U-Net; **(B)** Structure of modified U-Net.

The U-Net model is a common semantic segmentation network with an “U” shape (Ronneberger et al., 2015; Zhou et al., 2020) for image segmentation (Figure 3A). The left part of the network, the “encoder,” was repeatedly sampled by two convolution layers and one down-sampling layer. The right part of the network, the “decoder,” was connected by a deconvolution layer to the feature graph output by the “encoder.” Then deconvolution was performed two times. Finally, the channels output the desired number of categories through a 1 × 1 convolution operation. Based on the original U-Net model, a modified U-Net model was proposed in this research (Figure 3B) by reducing the number of convolution layers to accelerate the running time. Moreover, the inception module was used to increase the generalization ability and learning ability of the neural network. In the down-sampling layer, the inception module was used to replace the ordinary 3 × 3 convolutional layer, and a 1 × 1 convolution layer was connected after the inception module to reduce the input information and the model size.

**Figure 4:**
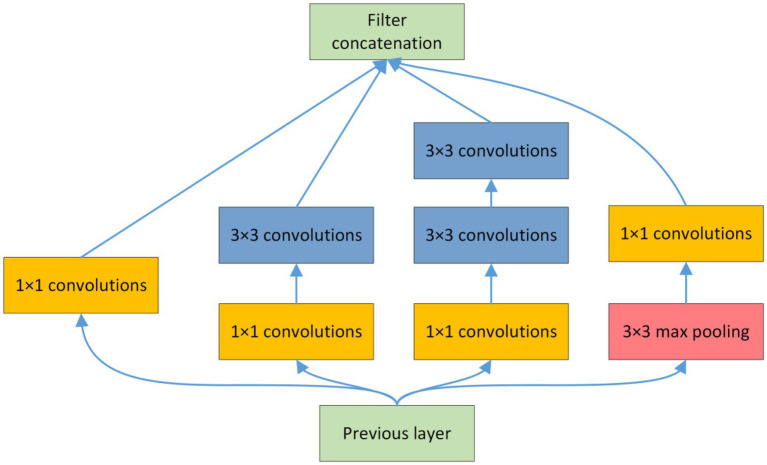
Structure of the inception module: **(A)** Training and validation loss; **(B)** Training and validation accuracy.

Depth and width are important parameters that affect convolutional neural networks. While increasing the network depth and width, the inception module also solves the problem of too many parameters and reduces the amount of parameter calculation (Szegedy et al., 2015). The inception module used in this study is shown in Figure 4. Features of cotton fields of different scales were extracted using 1 × 1 and 3 × 3 convolutional layers. Therefore, the multiscale inception module is suitable for determining characteristics of the multimorphic, multiscale, and random distribution of residual films in pre-sowing cotton fields. In the inception module, the fusion of different scales and functional branches was realized through the construction of cascade relationships, and then the fusion of multiscale image features was realized.

### Training for residual film detection

The deep learning model training hardware consisted of an Intel(R) Xeon(R) W-2223 CPU @ 3.60 GHz and 128 GB memory, and an NVIDIA GeForce RTX 3090 Graphics with 24 GB memory. The software environment was Windows 10, CUDA 11.2, CUDNN 8.1.1, Python 3.8, and TensorFlow-GPU 2.5.

To simulate the actual application scenario, the hardware and software for the residual film pollution evaluation included an Intel (R) Xeon (R) CPU E3-1230 V2 @ 3.30 GHz, without GPU acceleration, 16 GB memory, Windows 10 operating system, Python 3.7, and TensorFlow-CPU 2.3.

In this study, in the segmentation of residual films, a pixel is either classified as a residual film pixel or not. Similar to other binary classification networks, the “sparse categorical cross-entropy” function was used as the loss function. The neural networks were trained with a gradient descent method. The Adam optimizer algorithm was used to optimize the network, and the initial learning rate was 0.001. The batch of the training set was 6. During the iterative training process, changes in accuracy and loss were recorded, while only the best model was saved. When the number of training iterations reached 55, the training process converged and stopped.

### Network segmentation performance evaluation

In this study, the accuracy, F1-score, and mean IOU (MIOU) were used to assess the segmentation performance. The F1-score represents the combined results of precision and recall. The segmentation time and parameters of model were used to assess the segmentation speed and size, respectively.


(1) $$\mathrm{Accuracy}=\frac{\mathrm{TP}+\mathrm{TN}}{\mathrm{TP}+\mathrm{FP}+\mathrm{TN}+\mathrm{FN}}\times 100\%$$

(2) $$\mathrm{Precision}=\frac{\mathrm{TP}}{\mathrm{TP}+\mathrm{FP}}\times 100\%$$

(3) $$\mathrm{Recall}=\frac{\mathrm{TP}}{\mathrm{TP}+\mathrm{FN}}\times 100\%$$

(4) $$F1-\mathrm{score}=\frac{2\times \mathrm{Precision}\times \mathrm{Recall}}{\mathrm{Precision}+\mathrm{Recall}}\times 100\%$$

(5) $$\mathrm{IOU}=\frac{1}{2}\times \left(\frac{\mathrm{TP}}{\mathrm{TP}+\mathrm{FP}+\mathrm{FN}}+\frac{\mathrm{TN}}{\mathrm{TN}+\mathrm{FN}+\mathrm{FP}}\right)\times 100\%$$

Where TP is true positive, TN is true negative, FP is false positive, and FN is false negative.

### Evaluation of residual film pollution

The residual film coverage rate was used as the evaluation index of residual film pollution. For images with a size of M × N, the residual film coverage rate L is the ratio of the total number of residual film pixels [p (x, y) =1] to the total number of pixels in the image (Equation 6).


(6) $$L=\frac{\sum_{x=1,y=1}^{M,N}p\left(\mathrm{x,y}\right)}{M\times N}\times 100\%$$

To test the accuracy of the modified UNet in residual film pollution evaluation, the L values of 60 images were calculated. Then, the relationship between the predicted residual film coverage rate (L_1_) and true residual film coverage rate (L_2_) was evaluated by regression analysis. The coefficient of determination (R^2^), root mean square error (RMSE), and mean relative error (MRE) were selected as the evaluation indexes.


(7) $$R^{2}=1-\frac{\sum_{i=1}^{N}{\left(L_{2}-L_{1}\right)}^{2}}{\sum_{i=1}^{N}{\left(L_{2}-\bar{L_{2}}\right)}^{2}}$$

(8) $$\mathrm{RMSE}=\sqrt{\frac{1}{N}\overset{N}{\underset{i=1}{\sum }}{\left(L_{1}-L_{2}\right)}^{2}}$$

(9) $$\mathrm{MRE}=\frac{1}{N}\overset{N}{\underset{i=1}{\sum }}\frac{\left|L_{1}-L_{2}\right|}{L_{2}}\times 100\%$$

Where L_1_ and L_2_ are the i-th predicted and true L values from N data, respectively.

## Results

### Training process of the modified U-Net

**Figure 5:**
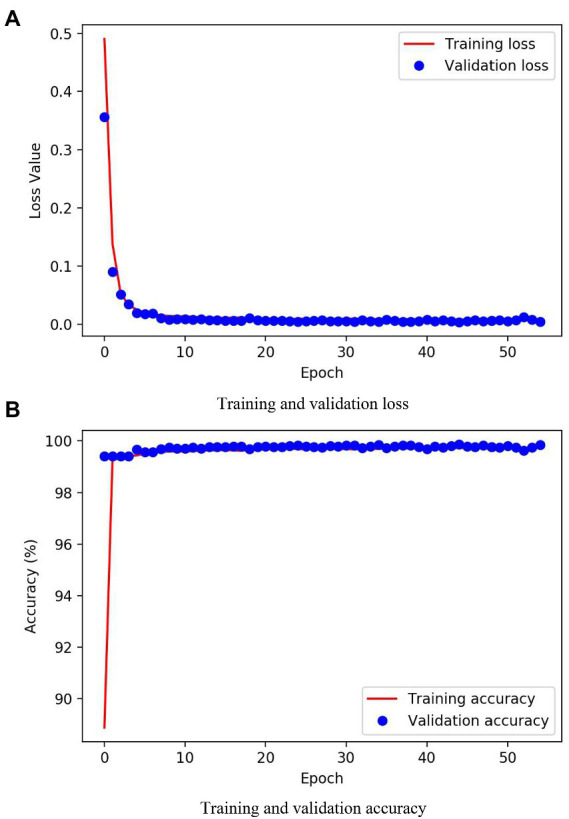
Loss and accuracy changes during training: **(A)** Training and validation loss; **(B)** Training and validation accuracy.

Figure 5 shows the change in loss and accuracy on the training and validation sets as the number of iterations increases during model training. The changes in the loss and accuracy of the training and validation sets showed the same trend. The loss value dropped first and then remained stable, and the accuracy value rose first and then remained stable. After approximately 10 epochs of training, both loss and accuracy remained stable. Furthermore, there was no significant difference in the previous loss values and the accuracy of the training and validation sets, so there was no model over-fitting. After iteratively training the model for 55 epochs, both the loss value and the accuracy converged, indicating that the model achieved good training results. After the model training stage, the loss and accuracy of the validation set were 0.0037 and 99.85%, respectively.

### Residual film segmentation results

#### Segmentation results of different models

The modified U-Net model was compared with the state-of-the art methods such as SegNet, FCN, and U-Net. The segmentation results of different models are shown in Table 2. The results showed that the modified U-Net model had the best performance and prediction accuracy on the test set. The accuracy of the modified U-Net was 99.72%, which was 0.25, 0.04, and 0.03% higher than that of SegNet, FCN, and U-Net, respectively. The F1-score of the modified U-Net model was 85.59%, which was 14.35, 2.91, and 1.83% higher than that of SegNet, FCN, and U-Net, respectively. The MIOU of the modified U-Net model was 87.53%, which was 10.02, 2.23, and 1.39% higher than that of SegNet, FCN, and U-Net, respectively. In terms of segmentation speed, the average segmentation time per image of the modified U-Net model was 192.50 ms, the minimum parameters of model were 3.14 × 10^6^ and was approximately 1/10 of that of the original U-Net model. Therefore, the modified U-Net model could improve the accuracy and speed of residual film segmentation, which facilitates the rapid and accurate identification of residual film.

**Table 2:** Segmentation results of different models.

Model	Accuracy (%)	F1-score (%)	MIOU (%)	Time (ms)	Parameters (10^6^)
SegNet	99.47	71.24	77.51	251.33	31.82
FCN	99.68	82.68	85.3	204.83	26.37
U-Net	99.69	83.76	86.14	245.17	31.06
Modified U-Net	99.72	85.59	87.53	192.50	3.14

#### Residual film segmentation results in different weather conditions

**Figure 6:**
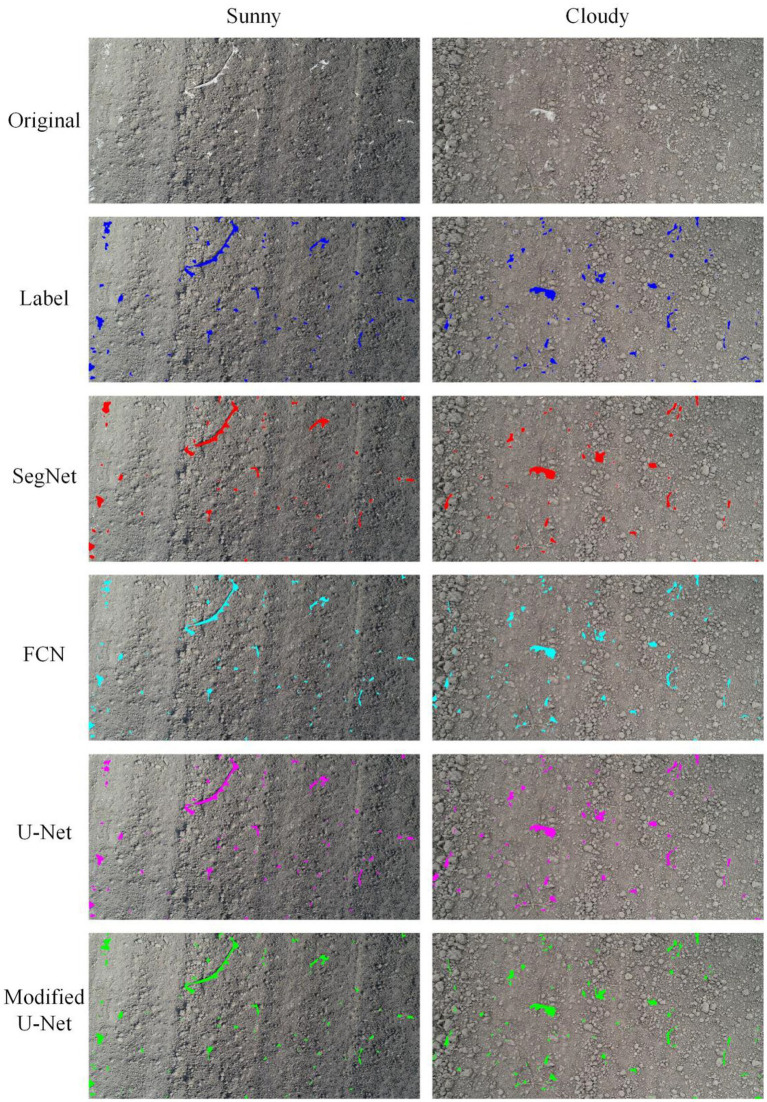
Residual film segmentation results under different weather conditions.

**Figure 7:**
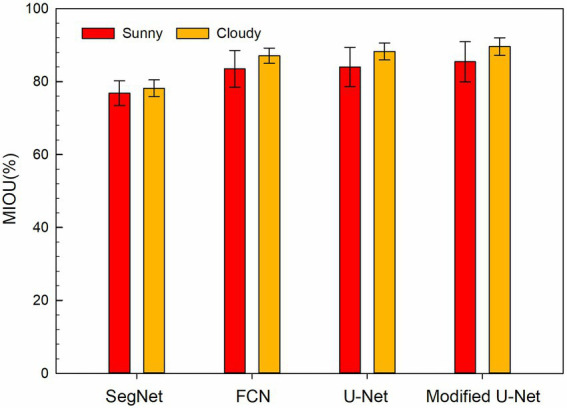
MIOU of different models under different weather conditions.

To study the influence of different weather conditions on the segmentation results. The segmentation results of cotton field images acquired in sunny and cloudy weather were compared (Figure 6). The results showed that no matter which model was used, the segmentation performance on images acquired on cloudy days was better than that on sunny days. Figure 7 shows the MIOU of different models based on the images acquired in different weather conditions. It showed that under the same weather conditions, the SegNet model had the worst segmentation performance, followed by the FCN and U-Net models. The modified U-Net model had the optimal performance, with MIOU reaching 85.44 and 89.63% on sunny and cloudy days, respectively.

#### Segmentation of images acquired at different heights

**Figure 8:**
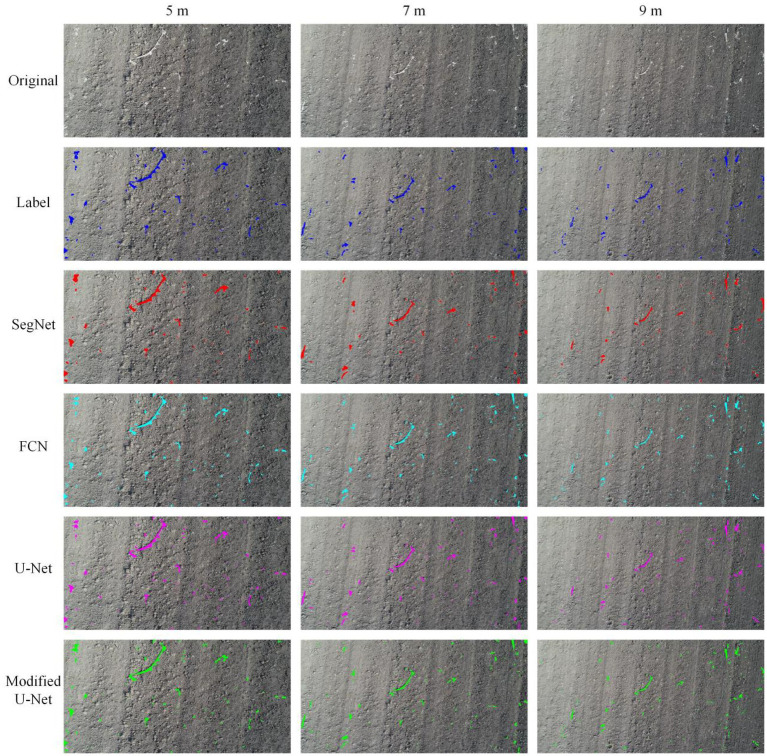
Residual film segmentation results of images acquired at different heights.

**Figure 9:**
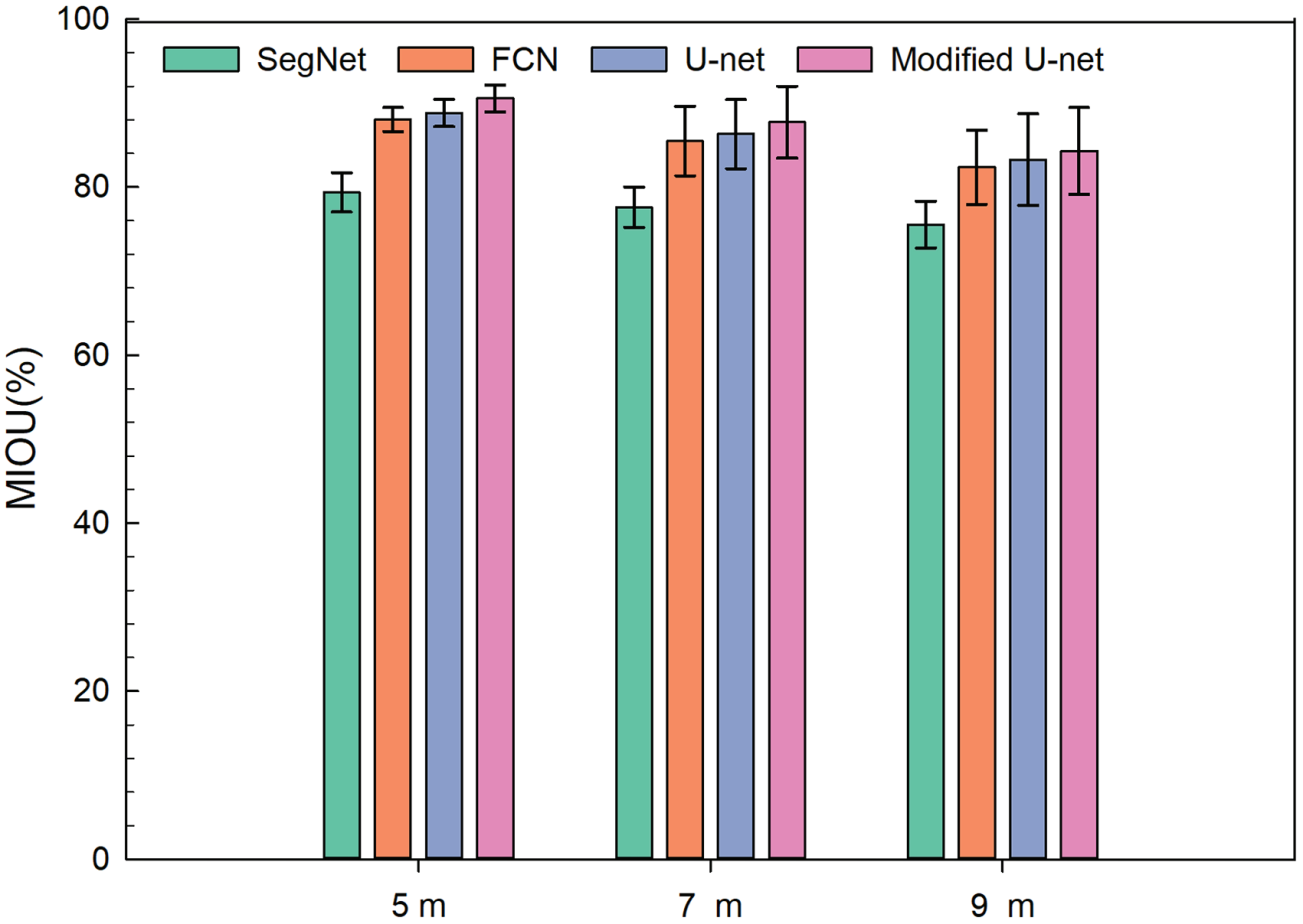
MIOU of different models based on the images acquired at different heights.

To study the effect of different image-acquiring height on the residual film segmentation results, the segmentation results of images acquired at the heights of 5, 7, and 9 M were compared (Figure 8). The results showed that the segmentation performance gradually decreased with the increase of height. Figure 9 shows the MIOU of different models based on the images acquired at different heights. The results showed that among the models, the SegNet model had the worst identification results at the same heights, followed by the FCN and U-Net models. The modified U-Net model had the optimal results, and its MIOU reached 90.55, 87.72, and 84.32% at 5, 7, and 9 M, respectively.

### Residual film pollution evaluation results

**Figure 10:**
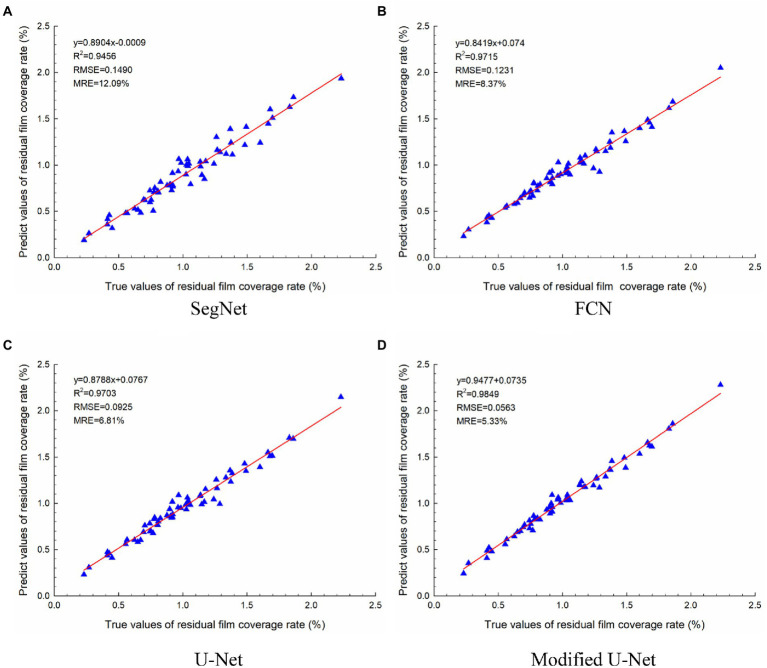
Regression analysis results of the UAV images-based evaluation and manual evaluation: **(A)** SegNet; **(B)** FCN; **(C)** U-Net; **(D)** Modified U-Net.

The regression analysis results of the UAV images-based evaluation and manual evaluation of different models are shown in Figure 10. The regression result of the modified U-Net model was slightly better than that of the other models, with a regression equation of y = 0.9477x + 0.7305. The R^2^, RMSE, and MRE were 0.9849, 0.0563, and 5.33%, respectively. Moreover, it was found that the intercept of the regression equations of different models was positive.

**Figure 11:**
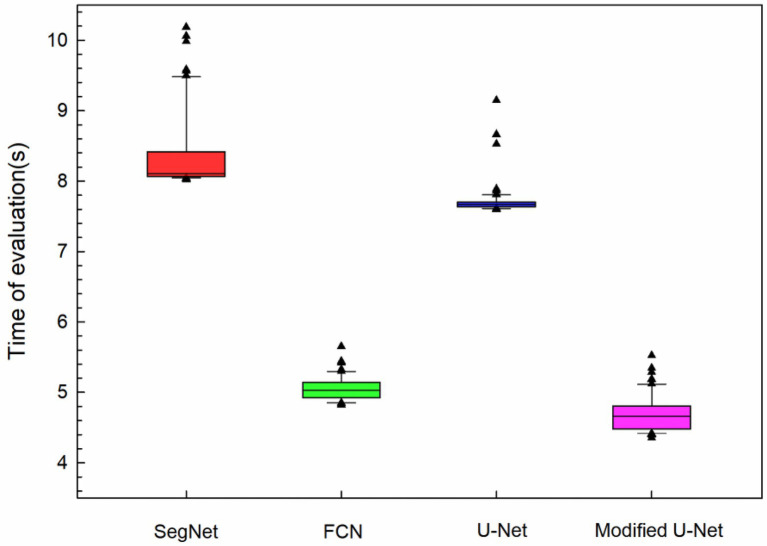
Time required by different models for residual film evaluation on the CPU.

The average evaluation time for 60 images in the test set on the CPU were statistically analyzed, and it was found that the evaluation time was slightly different. The time required to evaluate residual film pollution on the CPU is shown in Figure 11. It was found that the modified U-Net model had a minimum average evaluation time of 4.85 s, which was 41.07% less than the evaluation time of the U-net model.

## Discussion

This study identified residual film and evaluated the residual film pollution in cotton fields before sowing using low-altitude UAV imaging and deep learning. Based on the traditional U-Net model, a residual film semantic segmentation model with a modified U-Net model structure was proposed. This model could effectively segment the residual film from UAV images, the MIOU of the residual film recognition results reached 87.53%, which was 16.28 percentage points higher than the residual membrane pixel block identification method (Zhai et al., 2022). In this study, the residual film coverage rate was used to evaluate residual film pollution, and a rapid and accurate evaluation of residual film pollution was achieved based on the residual film semantic segmentation results. The results showed that the R^2^ of the modified U-Net model was 0.9849, the RMSE was 0.0563, the MRE was 5.33%, and the average evaluation time per image was 4.85 s on the CPU. These results indicate that the modified U-Net model can rapidly and accurately evaluate residual film pollution.

The residual film pollution evaluation method proposed in this study was mainly designed to identify residual films from the surface of cotton fields before sowing and to evaluate the degree of residual film pollution based on the proportion of residual films’ pixels. In this study, a multi classification neural network model was used to identify residual film, soil, straw, etc. Due to the surface of cotton fields includes residual film, soil, straw, drip irrigation belts, etc., it is very difficult to label each item one by one by pixel. Therefore, in the labelling process, only residual films (1) were manually labelled one by one, and soil, straw, and other items were marked as non-residual films (0). As the surface of the residual film attached to soil, the reflection of soil block and other reasons, resulting the existence of false positive (FP) and false negative detections (FN) in this study. The FP represents the segmentation model mistakenly identifies soil, straw and other samples as residual film samples; the FN represents the segmentation model mistakenly identifies residual film samples as soil, straw, etc.

This study proposed a model for residual film semantic segmentation based on a modified U-Net model. The image segmentation in this study is a binary classification, including identification and classification of residual films and non-residual films. Therefore, the feature extraction of the traditional U-Net model was simplified in this study to reduce the number of parameters and speed up the computation. Moreover, the multiscale feature extraction inception module was introduced to achieve accurate segmentation of residual films of different sizes by fusing multiscale image features. This modified network model may not perform as well on other more complex images but outperforms several traditional semantic segmentation models, including U-Net, SegNet, and FCN.

This study compared the identification performance on sunny and cloudy days and found that the identification performance on cloudy days was better than that on sunny days. This may be due to that the reflection of soil blocks causing them to be misjudged as residual films on sunny days. In addition, by comparing the effect of different image-acquiring height on the residual film segmentation, it was found that the lower the height is, the better the residual film segmentation effect. This may be due to that images acquired at lower heights have higher definition. However, when the height was too low, wind from the UAV’s rotor could blow away residual films, affecting the residual film pollution evaluation. Therefore, in practical applications, the height of UAV should be considered while ensuring image definition.

The residual film pollution evaluation method in this paper has application value for the control of residual film pollution. This evaluation system can achieve a rapid and accurate evaluation of residual film pollution. Moreover, rapid evaluation of the degree of residual film pollution can provide some reference for the objective evaluation of the seeding suitability of cotton fields during the spring sowing stage. In addition, this study also provides the theoretical support for the detection of residual film pollution in cotton field plough layer using UAV imaging, the rapid prediction of residual film pollution in cotton field plough layer can be realized by studying the residual film pollution correlation between the surface and plough layer. Compared to manual sampling to monitor residual film pollution, the approach in this study saves manpower and reduces time costs.

## Conclusion

In this paper, residual film pollution images in pre-sowing cotton fields were collected by UAV imaging system. The more suitable residual film segmentation model was built by modified U-Net model. Finally, the residual film pollution was evaluated based on residual film coverage rate. Through the analysis of the test results, it was found that:

(1) The modified U-Net model was proposed by simplifying the U-Net model and introducing an inception module, which can realize the accurate segmentation of residual film from cotton fields before sowing. The MIOU of segmentation reached 87.53%.
(2) The identification performance on cloudy days was better than that on sunny days. The identification performance of residual films gradually decreased with increasing image-acquiring height.
(3) The modified U-Net model outperformed other models in residual film pollution evaluation, with R^2^ of 0.9849, RMSE of 0.0563, MRE of 5.33% and the average evaluation time per image of 4.85 s on the CPU.
(4) This study provides a theoretical reference for further development of evaluation technology and equipment for residual film pollution based on UAV imaging.

## Data availability statement

The original contributions presented in the study are included in the article/supplementary material; further inquiries can be directed to the corresponding author.

## Author contributions

ZZ: methodology, model design, data analysis, and original manuscript writing. FQ: data collection and data analysis. QM: data analysis and revision. JY: data collection and revision. HW: data collection. XC and RZ: methodology, editing, revision, supervision, and funding. All authors contributed to the article and approved the submitted version.

## Funding

The authors gratefully acknowledge the financial support provided by the National Natural Science Foundation of China (32060412), the High-level Talents Research Initiation Project of Shihezi University (CJXZ202104), the earmarked fund for China Agriculture Research System (CARS-15-17), and the Graduate Education Innovation Project of Xinjiang Autonomous Region (XJ2022G082).

## Conflict of interest

The authors declare that the research was conducted in the absence of any commercial or financial relationships that could be construed as a potential conflict of interest.

The reviewer GY declared a shared affiliation with the authors to the handling editor at the time of review.

## Publisher’s note

All claims expressed in this article are solely those of the authors and do not necessarily represent those of their affiliated organizations, or those of the publisher, the editors and the reviewers. Any product that may be evaluated in this article, or claim that may be made by its manufacturer, is not guaranteed or endorsed by the publisher.
